# Serotypes and Vaccine Coverage of *Streptococcus Pneumoniae* Colonization in the Nasopharynx of Thai Children in Congested Areas in Chiang Mai

**DOI:** 10.3390/pathogens9120988

**Published:** 2020-11-26

**Authors:** Anchalee Wangirapan, Satja Issaranggoon na Ayuthaya, Wasan Katip, Nongyao Kasatpibal, Raktham Mektrirat, Usanee Anukool, Peninnah Oberdorfer

**Affiliations:** 1 Division of Infectious Diseases, Department of Pediatrics, Faculty of Medicine, Chiang Mai University, Chiang Mai 50200, Thailand; cherry_cmu@hotmail.com (A.W.); satjaiss@gmail.com (S.I.n.A.); 2 Department of Pharmaceutical Care, Faculty of Pharmacy, Chiang Mai University, Chiang Mai 50200, Thailand; 3 Epidemiology Research Group of Infectious Disease (ERGID), Chiang Mai University, Chiang Mai 50200, Thailand; nongyao.ka@cmu.ac.th (N.K.); Raktham.m@cmu.ac.th (R.M.); usanee.anukool@cmu.ac.th (U.A.); 4 Division of Nursing Science, Faculty of Nursing, Chiang Mai University, Chiang Mai 50200, Thailand; 5 Department of Veterinary Bioscience and Veterinary Public Health, Faculty of Veterinary Medicine, Chiang Mai University, Chiang Mai 50200, Thailand; 6 Division of Clinical Microbiology, Department of Medical Technology, Faculty of Associated Medical Sciences, Chiang Mai University, Chiang Mai 50200, Thailand

**Keywords:** *Streptococcus pneumoniae*, serotypes, nasopharynx, children, colonization, pneumococcal vaccine

## Abstract

*Streptococcus pneumoniae* causes around 10% of all deaths in children younger than five years of age. This study aimed to examine the serogroups/serotypes of *S. pneumoniae* colonization and vaccine serotype coverage of this organism among Thai children. Nasopharyngeal swabs of children less than or equal to 15 years of age were obtained in congested areas in Chiang Mai from 1 February 2013 to 1 August 2013. The serotyping of *S. pneumoniae* isolates was performed using the ImmuLex™ kit and the vaccine serotype coverage for this organism was evaluated. A total of 292 children were enrolled. One hundred and thirty children (44.5%) had nasopharyngeal colonization with *Streptococcus pneumoniae*. Eighty-seven (66.9%) isolates were from children younger than five years of age, seventeen (13.1%) were from children aged 6–10 years, and twenty-six (20%) were from children aged 11–15 years. The five most common serogroups/serotypes isolated were 6 (6A, 6B, 6C) (46.1%), 23 (23F, 23A, 23B) (14.6%), 19 (19F, 19A, 19B, 19C) (8.5%), 15 (15F, 15A, 15B, 15C) (6.9%), and 14 (6.1%). Vaccine serotype coverages in pneumococcal conjugate vaccines (PCV):PCV7, PCV10, and PCV13 were 79.1%, 83.6%, and 85.9%, respectively. There were significant increases in coverage between PCV7 and PCV10 (from 79.1% to 83.6%, *p* < 0.001), PCV7 and PCV13 (from 79.1% to 85.9%, *p* < 0.001), and PCV10 and PCV13 (from 83.6% to 85.9%, *p* < 0.001). The majority of pneumococcal serogroup/serotype colonization in the nasopharynx of Thai children in the studied areas was included in the current licensed pneumococcal conjugated vaccines (PCVs). PCV vaccination should be considered for high-risk children to reduce the incidence of invasive pneumococcal disease among Thai children.

## 1. Introduction

*Streptococcus pneumoniae* is a gram-positive bacterium that can cause bloodstream infections in both immunocompetent and immunocompromised patients. *S. pneumoniae* bacterial infection complications include meningitis, arthritis, and heart diseases. *S. pneumoniae* bacteremia is known as invasive pneumococcal disease (IPD) [1].

Before the implementation of the pneumococcal conjugated vaccine (PCV), the incidence of IPD in the United States among children younger than five years of age was approximately 17,000 cases per year. This included 700 cases of meningitis and 200 deaths [1]. When the United States added the PCV to the vaccination program, the incidence of IPD decreased by 60 to 90 percent in children younger than two years of age [2,3,4,5,6,7].

The polysaccharides capsule on the outer surface of pneumococci that protects the bacteria from phagocytosis is the most important virulence factor [8]. The disease invasiveness classification and pneumococcal vaccine formation are based on a high heterogeneity of capsular polysaccharides [9]. To date, more than 90 pneumococcal serotypes have been identified [10], and the prevalence of different serotypes among colonization and diseases has been described [11].

Recently, there are three types of PCV available on the market: PCV7, PCV10, and PCV13. PCV7 was licensed in 2000 and contains seven serotypes which cause IPD in North America (4, 6B, 9V, 14, 18C, 19F, and 23F). In 2008, PCV10 was licensed in Canada, Australia, and European countries. It contains 10 serotypes (1, 5, 7F, and all serotypes in PCV7). PCV13, which contains 13 serotypes (3, 6A, 19A, and all serotypes in PCV10), was licensed in Chile and European countries in 2009. However, since pneumococcal conjugated vaccines were developed, only a few countries have added PCV to their national immunization programs. In October 2006, the World Health Organization (WHO) made a recommendation for the serotype composition of pneumococcal conjugated vaccines to be used in resource-poor developing countries [12]. IPD serotypes vary according to geographic region and year. Thus, regional studies are necessary to assist in the decision of developing a new vaccine [13,14].

Studies have shown that there were seven main serotypes which commonly caused IPD, i.e., 1, 5, 6A, 6B, 14, 19F, and 23F [15,16]. In developing countries, serotypes 1, 5, 6A/6B, and 14 were found to be the main cause of IPD. As serotypes 1, 5, and 6A are not in the PCV7, use of PCV7 could cause changes in serotype causing IPD, namely “replacement strains” [5,17,18,19,20,21,22]. In Australia, USA, Europe, and Asia Pacific, the incidence of serotype 19A was increasing [5,17,18,21,22]. The common serotypes found in Southeast Asia were 19F, 23F, 14, 6B, 1, and 19A [23]. In Thailand, studies found that common pneumococcal serotypes in children younger than five years of age were 6B, 23F, and 14, and in children older than five years of age and adults, 23F, 19F, and 6B were common [24,25,26,27,28,29,30,31,32,33]. Serotype 19A is hardly found in Thailand. Phongsamart et al. [25] reported that the pneumococcal serotypes causing IPD in children younger than five years of age in Thailand in 2000–2005 were 6B (27.8%), 23F (20%), 14 (10.4%), and 19F (9.6%); no change in the distribution of serotypes was reported, except serotype 14 which seemed to increase in 2005 [25]. Rates of serotype 6A and 19A, which are in PCV13, were estimated to be 5.2%, and no serotype 3 was found. Suwanpakdee et al. [27] found that pneumococcal serotypes causing IPD in patients aged 0–18 years in the Phramongkutklao Hospital from 2004–2008 were 6B (6.4%), 14 (4.3%), 15 (2.1%), 19F (2.1%), and 23A (2.1%) [27]. However, this study did not find serotypes 3, 6A, and 19A.

Many studies showed that children who lived in childcare centers were nasopharyngeal carriers of pneumococci [34,35,36,37,38,39]. The data from a 1999 surveillance in a Portuguese day care center indicated that the carriage rate of *S. pneumoniae* has increased continuously since 1996, from 47% to 63% over 4 years [36]. Sá-Leão et al. found in a one-year longitudinal study with 11 samplings of nasopharyngeal carriage of pneumococci among 47 children who attended a single day care center that 61.4% contained pneumococci [38].

The immune system of children, which is not fully developed, and poor hygiene can increase risk of colonization and infection with *S. pneumoniae* [34,35,36,37,38,39]. According to our knowledge, there are epidemiological data on pneumococcal colonization among children in congested areas in northern Thailand. This study was conducted to examine pneumococcal serotypes among Thai children in congested areas in Chiang Mai city, the capital of the northern region and Thailand’s second largest province, using epidemiological data.

## 2. Results

From 1 February 2013 to 1 August 2013, 292 children who lived in congested areas were recruited into this study. Of these, 52.1% were males. Most children were in the 11–15 years old age group (42.8%), followed by the less than 5 years old age group and the 6–10 years old age group (39.0% and 18.2%, respectively). The mean age was about 8 years old. The mean weight was approximately 26 kg. Approximately 1.4% of all children had chronic lung disease and congenital heart disease.

### 2.1. Nasopharyngeal Colonization

*S. pneumoniae* nasopharyngeal colonization was found in 44.5% (130/292) of children living in congested areas in Chiang Mai. Among bacteria and fungi detected in the childrens’ nasopharyngeal swabs (Table 1), *S. pneumoniae* was the second-most common bacterium found. *Moraxella catarrhalis* was the most frequently detected bacterial species in this study (58.9%), followed by *S. pneumoniae* (44.5%), *Corynebacterium spp*. (35.9%), *Staphylococcus aureus* (29.8%), and Coagulase-negative *Staphylococcus* (27.4%). Type B *Haemophilus influenzae* was not found in this study but other types of *H. influenzae* were found in 16.1% of the children. Other bacterial species found are listed in the footnote of Table 1. In addition, 3.4% of the organisms were found to be fungi.

### 2.2. Antimicrobial Susceptibility

Antimicrobial susceptibility of *S. pneumoniae, H. influenzae, M. catarrhalis*, and *S. aureus* to 13 antimicrobial agents are shown in Table 2. All *S. pneumoniae* isolates were susceptible to levofloxacin. However, approximately 69.2% and 61.5% of the isolates were susceptible to clindamycin and erythromycin, and only 16.9% and 10.8% were susceptible to penicillin with the oxacillin disk diffusion method and co-trimoxazole. The susceptibility of *S. pneumoniae* to cefotaxime was not assessed since disk diffusion testing of cephalosporin was found unreliable [35,36,37,38,39,40]. The susceptibility rates of *H. influenzae* and *M. catarrhalis* to cefotaxime and ciprofloxacin were 100%. All *S. aureus* isolates were susceptible to clindamycin, vancomycin, and fusidic acid. Approximately 98.6% of *S. aureus* isolates were susceptible to oxacillin; thus, 1.4% were identified as methicillin-resistant *S. aureus* (MRSA).

### 2.3. Serotypes of Nasopharyngeal Pneumococcal Colonization

A total of 130 pneumococcal-colonized children were detected from 292 children in congested areas in Chiang Mai. Eighty-seven (66.9%) *S. pneumoniae* isolates were from children younger than five years of age, seventeen (13.1%) were from children aged 6–10 years old, and twenty-six (20%) were from children aged 11–15 years.

**Figure 1:**
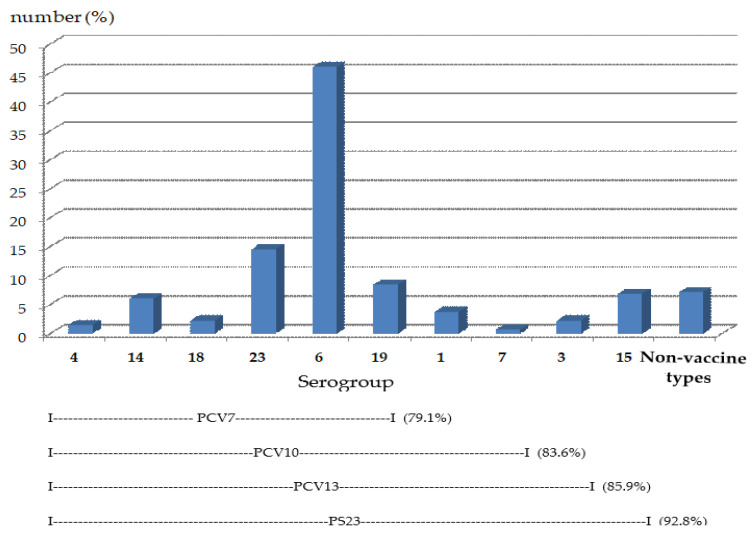
Overall serotype distribution of pneumococcal isolates. Note: “Non-vaccine types” defined as *S. pneumoniae* serogroups/serotypes not belonging to the vaccine serogroups/serotypes.

The five most common serogroups/serotypes isolated were 6 (6A, 6B, 6C) (46.1%), 23 (23F, 23A, 23B) (14.6%), 19 (19F, 19A, 19B, 19C) (8.5%), 15 (15F, 15A, 15B, 15C) (6.9%), and serotype 14 (6.1%) (Table 3). The serotype coverage was anticipated at 79.1% for PCV7 (serotypes 4, 9V, 14, 19F, 23F, 18C, and 6B), 83.6% for PCV10 (PCV7 serotypes plus serotypes 1, 5, and 7F), 85.9% for PCV13 (PCV7 serotypes plus serotypes 1, 5, 7F, 3, 6A, and 19A), and 92.8% for the 23-valent pneumococcal polysaccharide vaccine (serotypes 1, 2, 3, 4, 5, 6B, 7F, 8, 9N, 9V, 10A, 11A, 12F, 14, 15B, 17F, 18C, 19A, 19F, 20, 22F, 23F, and 33F) (Figure 1). There were significant differences in serotype coverage between PCV7 and PCV10 (from 79.1% to 83.6%, *p* < 0.001), PCV7 and PCV13 (from 79.1% to 85.9%, *p* < 0.001), and PCV10 and PCV13 (from 83.6% to 85.9%, *p* < 0.001).

Using a univariate analysis by logistic regression, the significant risk factors associated with *S. pneumoniae* colonization included a history of upper respiratory tract infection one month prior to specimen collection (OR = 49.20; 95% CI 6.68–362.15; *p* = 0.001), abnormal skin lesions on physical examination (OR = 7.22; 95% CI 3.20–16.26; *p* = 0.001), and upper respiratory tract infection observed at nasopharyngeal specimen-collection time (OR = 0.29; 95% CI 0.09–0.99; *p* = 0.048).

Using a multivariate analysis by logistic regression, the risk factors associated with *S. pneumoniae* colonization were upper respiratory tract infection one month before collection of specimen (OR = 68.91; 95% CI 6.48–732.87; *p* = 0.001) and abnormal skin lesions (OR = 4.01; 95% CI 1.51–10.64; *p* = 0.005) (Table 4).

## 3. Discussion

The percentage of nasopharyngeal colonization of *S. pneumoniae* found in this study was 44.5%, which was greater than the colonization rates reported from developed countries (21%) [40]. Eighty-seven isolates (66.9%) were from children less than five years of age, seventeen (13.1%) were from children aged 6–10 years, and twenty-six (20%) were from children aged 11–15 years. The five most common serogroups/serotypes isolated were 6 (6A, 6B, 6C) (46.1%), 23 (23F, 23A, 23B) (14.6%), 19 (19F, 19A, 19B, 19C) (8.5%), 15 (15F, 15A, 15B, 15C) (6.9%), and 14 (6.1%). The serotype coverages for PCV7, PCV10, and PCV13 were assumed to be 79.1%, 83.6%, and 85.9%, respectively. *S. pneumoniae* was susceptible to levofloxacin, erythromycin, and penicillin at 100%, 61.5%, and 16.9%, respectively. Risk factors associated with *S. pneumoniae* colonization were upper respiratory tract infection one month before specimen collection (OR = 68.91; 95% CI 16.48–732.87, *p* = 0.001) and abnormal skin lesions (OR = 4.01; 95% CI 1.51–10.64, *p* = 0.005). Upper respiratory tract infection commonly included sinusitis, otitis media, pharyngitis, and the common cold.

*Moraxella catarrhalis* was the most common bacterium found (58.9%). *S. pneumoniae* (44.5%) was the second most common organism found. *Staphylococcus aureus* including MRSA, coagulase-negative *Staphylococcus*, and non-type B *H. influenzae* were found in 29.8%, 27.4%, and 16.1% of all isolates, respectively. However, *H. influenzae* type B was not detected in this study.

The most common serogroups for *Streptococcus pneumoniae* nasopharyngeal colonization of children under the age of five years who lived in congested areas in Chiang Mai were 6, 23, 19, 15, and 14 (in rank order). In children aged 6–15 years the most prevalent serogroups were 6, 23, 19, and 3.

A recent study of the prevalence of *S. pneumoniae* among healthy children in Thailand was conducted in Nakhon Phanom province (the northeastern region) and Sa Kaeo province (the eastern region) [41]. This multi-country case-control study under the Pneumonia Etiology Research for Child Health Project (PERCH) reported nasopharyngeal colonization with *Streptococcus pneumoniae* in children aged 1–59 months (from January 2012–February 2014) of 62.5% in community control and 54.5% in cases (severe/very severe pneumonia cases). However, a lower percentage of colonizing isolates from cases and community control that were the serotypes included in PCV10 (70.0% and 61.8%, respectively) and PCV13 (76.7% and 67.9%, respectively) was observed [42].

The previous work conducted in Thailand reported a lower nasal *S. pneumoniae* carriage rate of 16% in children aged 2–10 years from four schools in three different districts in Phitsanulok province, the middle part of Thailand [41]. Resistances to clindamycin, erythromycin, and co-trimoxazole were found in 18.4%, 21.1%, and 78.9% of all isolates, respectively. The high rate of resistance to commonly prescribed antibiotics correlated to the results of this study while 30.8%, 38.5%, and 89.2% of all isolates were resistant to clindamycin, erythromycin, and co-trimoxazole, respectively [43,44]. A cross-sectional study in Iceland also reported the correlation of the carriage of drug-resistant pneumococci in children with risk factors such as recent antibiotic use, living in an area with high consumption of antibiotics, and use of co-trimoxazole [45].

A study in Peru revealed high rates of colonizing *S. pneumoniae* in healthy children, 92% (467/506) in 2009 and 89% (451/509) in 2011 [46]. In 2009, 23F, a serotype included in PCV7, was the only type identified as a persister, and 6A, 15B, and 19A were identified as recolonizer serotypes. In 2011, 6B and 7C were persister serotypes, and 13 was a frequent recolonizer serotype. The prevalence of nasopharyngeal carriage of *S. pneumoniae* serotypes among children aged 2–59 months in India was studied in Palwal District, Haryana, from December 2016 to July 2017, before the introduction of pneumococcal conjugate vaccines [47]. The colonization rate of *S. pneumoniae* was 74.7% and 54.5% among children with clinical pneumonia and community children, respectively. The prevalence of PCV13 vaccine-type colonization was similar between children with clinical pneumonia (31.9%) and community children (28.0%; *p* = 0.46). The most predominant serotypes were 6A, 6B, 14, 19A, 19F, and 23F, all of which are included in the PCV13 vaccine product. Antimicrobial resistance to at least one drug was similar between isolates from children with clinical pneumonia (66.1%) and community children (61.5%; *p* = 0.49), while resistance to at least two drugs was more common among isolates from children with clinical pneumonia than those from community children (25.8% vs. 16.4%; *p* = 0.08) [48]. The prevalence and distribution of *S. pneumoniae* serogroups/serotypes colonization varied widely among distinct geographic locations since it depends heavily on a range of host and environmental factors [48,49].

This study supports the results from previous studies reporting on pneumococcal isolates causing IPD in Thailand; the most common vaccine serotypes in children under the age of five years were 6B, 23F, and 14, and in children aged five years and above, 23F, 19F, and 6B were most common [23,24,25,26,27,28,29,30,31,32,33]. A study from Southeast Asian countries found that the top six (in rank order of frequency) pneumococcal serogroups/serotypes causing IPD were 19, 6, 23, 14, 1, and 3 [23]. This study, however, found that the most predominant serogroup that colonized in the nasopharynx of children was serogroup 6. Serogroup 19 ranked the third highest in the order of detected serogroups. According to a pneumococcal global serotype project, the most common global serotypes (in rank order) are 14, 6B, 1, 23F, 5, 19F, 6A, and 19A [15]. A review of published studies of IPD in North American, European, and Latin American children from 1995–1999 by Hausdorff [50] showed that, across the world, the most commonly found serogroups/serotypes that caused serious diseases and complication resulting in hospitalization were 1, 5, and 7 [50]. However, the present study found small numbers of serogroups 1 and 7, and none of serogroup 5. This different finding might be a result of the virulence of the serotypes. This work studied the serogroups/serotypes of *S. pneumoniae* that colonized in the nasopharynx, not the serotypes of clinical *S. pneumoniae* isolates that caused IPD. A review by Jauneikaite et al. [23] found that vaccine coverage of *S. pneumoniae* in Thailand was 44% for PCV7, 50% for PCV10, and 63% for PCV13 [24]. In this study, the serotype coverages were higher since the serotype coverages from PCV7, PCV10, PCV13, and 23-valent pneumococcal polysaccharide vaccine, were 79.1%, 83.6%, 85.9%, and 92.8%, respectively (Figure 1). The higher coverage rates of vaccine in this study might be due to the high rates of *S. pneumoniae* colonization in the studied population. In addition, this population was not given the pneumococcal vaccine prior to the study, so the serotypes not included in the vaccine (termed “replacement strains”) were low. Although the coverage rate by 23-valent pneumococcal polysaccharide vaccine seemed to be higher than that of the PCV, it could not decrease nasal colonization by *S. pneumoniae* [40]. Polysaccharide vaccines were also poorly immunogenic in children younger than two years of age, who had the highest incidence of invasive pneumococcal disease [51,52,53,54,55]. In this study, the percentage of non-vaccine type *S. pneumoniae* was low, so the existing vaccine formulation may be proper for children in Chiang Mai in order to prevent and control invasive disease.

This study indicated that at least 16.9% of *S. pneumoniae* isolates were susceptible to penicillin by using a 1-µg oxacillin disk screening test. Pongsamart et al. [25] found that 69.6% of pneumococcal isolates were resistant to penicillin. There was an increase of penicillin resistance from 58.3% in 2000 to 88.2% in 2005 [25]. Higher rates of penicillin resistance have been described in other countries. This was illustrated in a review of 685 *S. pneumoniae* isolates from 14 centers in 11 Asian countries that were collected from January 2000 to June 2001 [56]. Overall, 52% were resistant to penicillin, with rates of resistance as high as 74% in Vietnam [31,57,58,59,60]. In the United States, approximately 85% of pneumococci were susceptible to penicillin, 10% were intermediately resistant, and 5% were highly resistant [61,62]. In the present study, 1-µg oxacillin disk diffusion was used to test the susceptibility of *S. pneumoniae* to penicillin; the result “susceptible” was reported when the zone size was equal to or larger than 20 mm [63]. However, the penicillin resistance test was not performed in non-susceptible *S. pneumoniae* strains that presented zone sizes equal to or less than 19 mm by the minimum inhibitory concentration (MIC) test. Moreover, the resistance to penicillin and cephalosporin using the MIC test was not carried out in penicillin non-susceptible isolates. Hence, MIC tests of penicillin and cephalosporin should be further investigated.

This study is the first study to explore the prevalence and the serogroups/serotypes frequency of *S. pneumoniae* nasopharyngeal colonization among children who live in congested areas in Chiang Mai. Because nasopharyngeal colonization of *S. pneumoniae* could increase risk of IPD [64], the results reported here are useful for consideration of currently available vaccines. However, there were still some limitations in this study. Some *S. pneumoniae* isolates could not be identified at the serotype level due to the limitation in budget and test kit availability. Here, serotyping of *S. pneumoniae* was performed using the ImmuLex™ test (Statens Serum Institute Diagnostica, Hovedstaden, Denmark). This latex agglutination-based test was shown to identify 47.8% of *S. pneumoniae* in 67 normally sterile clinical samples (blood, cerebrospinal fluid (CSF), and pleural fluid) only to the level of serogroup (6, 7, 9, 10, 11, 12, 15, 19, 22, 23, or 33) [65]. Although there was no discrepancy between serogroups determined by the reference (PCR) and latex methods, the latex test did not allow the maximum discrimination (to the serotype level) [65]. Nevertheless, the main serotypes or serogroups that are included in the pneumococcal vaccine were identified in all strains isolated from children in this study. Therefore, vaccine serotype coverage reported in the present study might be overestimated. Nevertheless, immunologic cross-reactivity among serotypes in the same serogroup might result in cross-protection, such as 19F and 19A, and 6B and 6A [66,67,68,69,70].

## 4. Materials and Methods

This prospective cohort study was performed on children less than or equal to 15 years old in congested areas in Chiang Mai. These children are at risk of infection from *S. pneumoniae* since they live in congested areas and have recurrent infections.

Congested areas in this study are defined as dormitories and orphanages in Chiang Mai, Thailand. Data were collected from 1 February 2013 to 1 August 2013 (7 months). The data were analyzed for 5 months, and the study was completed in January 2014. Children aged less than or equal to 15 years were recruited in congested areas in Chiang Mai, including one dormitory and three orphanages. This study was approved by the ethics committee on human research of the Faculty of Medicine, Chiang Mai University (Research ID: 1267/Study Code PED-12-1267-EX). The caretakers of the children were informed of study details, and consent was asked from the participants in this study.

### 4.1. Study Population and Data Collection

#### 4.1.1. Sample Size Calculation

Power and sample size calculated by testing for the proportion of patients in the previous studies showed that the rate of nasopharyngeal carriers for *S. pneumoniae* was about 21% in developed countries and 90% in developing countries [71]. Since Thailand is a developing country, 90% of incidence was chosen to calculate the sample size.

The sample size was 138 children, so approximately 150 specimens were needed. However, a preliminary study showed that only 50% of nasopharyngeal swabs were positive; this study therefore performed nasopharyngeal swabs on 300 children in order to achieve the needed sample size.

#### 4.1.2. Data Collection

The data of each child were recorded, including age, sex, weight, height, ethnicity, address, immunization history, history of illness in the past one month, underlying disease, and medication.

### 4.2. Specimen Collection and Isolation of S. pneumoniae

A nasopharyngeal swab was obtained, plated directly onto 5% blood agar and chocolate agar, and then incubated at 35–37 °C with 5% CO_2_ atmosphere for 24–48 h. The typical colonies of *S. pneumoniae* with a zone of alpha-hemolysis (green) were selected for biochemical identification.

### 4.3. Bacterial Identification and Characterization

*S. pneumoniae* isolates were identified using Gram stain, catalase, optochin, and bile solubility tests [42]. Other recovered bacteria were also identified according to standard microbiological tests.

Antimicrobial susceptibility testing (AST) of *S. pneumoniae*, *H. influenzae*, *M. catarrhalis*, and *S. aureus* was performed using disk diffusion method based on the clinical and laboratory standards institute (CLSI) guideline [72].

The 1-μg oxacillin disk diffusion was used to test susceptibility of *S. pneumoniae* to penicillin. Serotyping of *S. pneumoniae* was performed using the ImmuLex™ test as described by the packaging inserts (Statens Serum Institute Diagnostica, Hovedstaden, Denmark).

### 4.4. Statistical Analysis

Statistical analysis was performed using Stata software, version 14 (Stata-Corp, College Station, TX, USA). Descriptive statistics were used to report the results. Mean (±standard deviation, SD) and median (range) were used for continuous data, while counts and percentages were used for nominal data. Continuous variables with normal distribution were compared by student t-test. All tests were two-sided, and statistical significance was set at a *p* value of ≤0.05. Univariate analysis was performed by logistic regression to compare categorical variables as appropriate. Furthermore, multivariate analysis by logistic regression was used to estimate the risk factors associated with *S. pneumoniae* colonization. The results of the logistic regression were expressed as the adjusted odds ratio (aOR) and 95% confidence interval (CI).

## 5. Conclusions

In conclusion, this study showed that the majority of pneumococcal serogroups/serotypes colonizing the nasopharynx of children who lived in congested areas were included in the current licensed PCV. Giving this vaccine to these high-risk children therefore appears to be useful and should be supported by the government. In the future, the prevalence of pneumococcal serogroups/serotypes of the post-PCV vaccination period should be investigated in order to identify changes in regional serotypes that may occur.

## Figures and Tables

**Table 1:** Total colonization.

Organisms	Number (%) (Total = 292)
Bacteria	
*Moraxella catarrhalis*	172 (58.9)
*Streptococcus pneumoniae*	130 (44.5)
*Corynebacterium* spp. (Diphtheroids)	105 (35.9)
*Staphylococcus aureus*	87 (29.8)
Coagulase-negative *Staphylococcus*	80 (27.4)
*Haemophilus influenzae* (not type B)	47 (16.1)
α-hemolytic streptococci	36 (12.3)
*Micrococcus* spp.	30 (10.3)
Non-hemolytic streptococci	26 (8.9)
*Neisseria* spp.	26 (8.9)
*Haemophilus parainfluenzae*	15 (5.1)
Others *	15 (5.1)
Fungi	10 (3.4)

* *Bacillus* spp. 9 (3.1), *Pseudomonas aeruginosa* 2 (0.7), *Klebsiella pneumoniae* 1 (0.3), *Enterobacter agglomerans* 1 (0.3), *Enterobacter cloacae* 1 (0.3), β-hemolytic streptococci (not group A, B, or D 1) (0.3).

**Table 2:** Antimicrobial susceptibility.

Antimicrobial Agents	Sensitivity (%)			
	*S. pneumoniae*	*H. influenzae*	*M. catarrhalis*	*S. aureus*
Chloramphenicol	-	87.2	97.6	-
Co-trimoxazole	10.8	46.8	42.4	96
Penicillin	16.9	-	-	-
Oxacillin	-	-	-	98.6
Ampicillin	-	68.1	94.2	-
Amoxicillin/clavulanate	-	95.7	100	-
Erythromycin	61.5	-	-	93.3
Clindamycin	69.2	-	-	100
Cefotaxime	-	100	100	-
Ciprofloxacin	-	100	100	-
Vancomycin	-	-	-	100
Fusidic acid	-	-	-	100
Levofloxacin	100	-	-	-

**Table 3:** Serotypes of pneumococcal isolates by age groups.

Serogroups/Serotypes	Number of *S. pneumoniae* Isolates Found in Children at Different Ages (%)			
	<5 Years	6–10 Years	11–15 Years	Total
**Vaccine Types**				
**1**	5 (3.8)	-	-	5 (3.8)
**3**	1 (0.7)	-	2 (1.5)	3 (2.3)
**4**	1 (0.7)	1 (0.7)	-	2 (1.5)
6 (6A, **6B**, 6C)	41 (31.5)	7 (5.4)	12 (9.2)	60 (46.1)
7 (**7F**, 7A, 7B, 7C)	-	-	1 (0.7)	1 (0.7)
**14**	7 (5.4)	1 (0.7)	-	8 (6.1)
15 (15F, 15A, **15B**, 15C)	9 (6.9)	-	-	9 (6.9)
18 (18F, 18A, 18B, **18C**)	2 (1.5)	1 (0.7)	-	3 (2.3)
19 (**19F**, **19A**, 19B, 19C)	7 (5.4)	-	4 (3.1)	11 (8.5)
23 (**23F**, 23A, 23B)	9 (6.9)	4 (3.1)	6 (4.6)	19 (14.6)
**Non Vaccine Types**				
13, 28 (28F, 28A)	1 (0.7)	-	-	1 (0.7)
21, 39	-	1 (0.7)	-	1 (0.7)
24 (24F, 24A, 24B), 31, 40	1 (0.7)	-	-	1 (0.7)
25 (25F, 25A), 38, 43, 44, 45, 46, 48	1 (0.7)	1 (0.7)	-	2 (1.5)
29, 34, 35 (35F, 35A, 35B, 35C), 42, 47 (47F, 47A)	2 (1.5)	1 (0.7)	1 (0.7)	4 (3.1)
Total	87 (66.9)	17 (13.1)	26 (20)	130 (100)

Note: Boldface indicates the serogroup/serotype included in pneumococcal vaccine. ( ) states serotypes within a serogroup.

**Table 4:** Risk factors associated with *S. pneumoniae* colonization (multivariate analysis).

Factor	Adjusted OR (95% CI)	*p*-Value
**History**		
Upper respiratory tract infection one month before collection of specimen	**68.91 (6.48–732.87)**	0.001 *
Eczema one month before collection of specimen	0.59 (0.01–26.07)	0.784
**Physical examination**		
Anemia	0.46 (0.09–2.45)	0.361
Tonsil enlargement	2.19 (0.74–6.52)	0.155
Dental caries	1.26 (0.55–2.87)	0.585
Abnormal skin lesions	**4.01 (1.51–10.64)**	0.005 *
**Comorbidity**		
Upper respiratory tract infection	0.77 (0.13–4.74)	0.780

Note: * *p* < 0.05.

## References

[1] Advisory Committee on Immunization Practices (2000). Preventing pneumococcal disease among infants and young children. Recommendations of the Advisory Committee on Immunization Practices (ACIP). MMWR Recomm Rep..

[2] Whitney C.G., Farley M.M., Hadler J., Harrison L.H., Bennett N.M., Lynfield R., Reingold A., Cieslak P.R., Pilishvili T., Jackson D. (2003). Active Bacterial Core Surveillance of the Emerging Infections Program Network. Decline in invasive pneumococcal disease after the introduction of protein-polysaccharide conjugate vaccine. N. Engl. J. Med..

[3] Centers for Disease Control and Prevention (CDC) (2005). Direct and indirect effects of routine vaccination of children with 7-valent pneumococcal conjugate vaccine on incidence of invasive pneumococcal disease—United States, 1998–2003. MMWR Morb. Mortal. Wkly. Rep..

[4] Poehling K.A., Talbot T.R., Griffin M.R., Craig A.S., Whitney C.G., Zell E., Lexau C.A., Thomas A.R., Harrison L.H., Reingold A.L. (2006). Invasive pneumococcal disease among infants before and after introduction of pneumococcal conjugate vaccine. JAMA.

[5] Centers for Disease Control and Prevention (CDC) (2008). Invasive pneumococcal disease in children 5 years after conjugate vaccine introduction—Eight states, 1998–2005. MMWR Morb. Mortal. Wkly. Rep..

[6] Hsu H.E., Shutt K.A., Moore M.R., Beall B.W., Bennett N.M., Craig A.S., Farley M.M., Jorgensen J.H., Lexau C.A., Petit S. (2009). Effect of pneumococcal conjugate vaccine on pneumococcal meningitis. N. Engl. J. Med..

[7] Pavia M., Bianco A., Nobile C.G., Marinelli P., Angelillo I.F. (2009). Efficacy of pneumococcal vaccination in children younger than 24 months: A meta-analysis. Pediatrics.

[8] Cherian T. (2007). WHO expert consultation on serotype composition of pneumococcal conjugate vaccines for use in resource-poor developing countries, 26–27 October 2006, Geneva. Vaccine.

[9] Watson D.A., Musher D.M. (1999). A brief history of the pneumococcus in biomedical research. Semin. Respir. Infect..

[10] Hausdorff W.P., Bryant J., Paradiso P.R., Siber G.R. (2000). Which pneumococcal serogroups cause the most invasive disease: Implications for conjugate vaccine formulation and use, part I. Clin. Infect. Dis..

[11] Bratcher P.E., Park I.H., Hollingshead S.K., Nahm M.H. (2009). Production of a unique pneumococcal capsule serotype belonging to serogroup 6. Microbiology.

[12] Brueggemann A.B., Peto T.E., Crook D.W., Butler J.C., Kristinsson K.G., Spratt B.G. (2004). Temporal and geographic stability of the serogroup-specific invasive disease potential of *Streptococcus pneumoniae* in children. J. Infect. Dis..

[13] Harboe Z.B., Benfield T.L., Valentiner-Branth P., Hjuler T., Lambertsen L., Kaltoft M., Krogfelt K., Slotved H.C., Christensen J.J., Konradsen H.B. (2010). Temporal trends in invasive pneumococcal disease and pneumococcal serotypes over 7 decades. Clin. Infect. Dis..

[14] Miller E., Andrews N.J., Waight P.A., Slack M.P., George R.C. (2011). Herd immunity and serotype replacement 4 years after seven-valent pneumococcal conjugate vaccination in England and Wales: An observational cohort study. Lancet Infect. Dis..

[15] Reinert R.R., Paradiso P., Fritzell B. (2010). Advances in pneumococcal vaccines: The 13-valent pneumococcal conjugate vaccine received market authorization in Europe. Expert Rev. Vaccines.

[16] Johnson H.L., Deloria-Knoll M., Levine O.S., Stoszek S.K., Freimanis H.L., Reithinger R., Muenz L.R., O’Brien K.L. (2010). Systematic evaluation of serotypes causing invasive pneumococcal disease among children under five: The pneumococcal global serotype project. PLoS Med..

[17] Lehmann D., Willis J., Moore H.C., Giele C., Murphy D., Keil A.D., Harrison C., Bayley K., Watson M., Richmond P. (2010). The changing epidemiology of invasive pneumococcal disease in aboriginal and non-aboriginal western Australians from 1997 through 2007 and emergence of nonvaccine serotypes. Clin. Infect. Dis..

[18] Hsu K.K., Shea K.M., Stevenson A.E., Pelton S.I. (2010). Massachusetts Department of Public Health. Changing serotypes causing childhood invasive pneumococcal disease: Massachusetts, 2001–2007. Pediatr. Infect. Dis. J..

[19] Weinberger D.M., Malley R., Lipsitch M. (2011). Serotype replacement in disease after pneumococcal vaccination. Lancet.

[20] Hanquet G., Kissling E., Fenoll A., George R., Lepoutre A., Lernout T., Tarragó D., Varon E., Verhaegen J. (2010). Pneumococcal serotypes in children in 4 European countries. Emerg. Infect. Dis..

[21] Roche P.W., Krause V., Cook H., Barralet J., Coleman D., Sweeny A., Fielding J., Giele C., Gilmour R., Holland R. (2008). Invasive pneumococcal disease in Australia; 2006. Commun. Dis. Intell. Q Rep..

[22] Ho P.L., Chiu S.S., Ang I., Lau Y.L. (2011). Serotypes and antimicrobial susceptibilities of invasive *Streptococcus pneumoniae* before and after introduction of 7-valent pneumococcal conjugate vaccine, Hong Kong, 1995–2009. Vaccine.

[23] Jauneikaite E., Jefferies J.M., Hibberd M.L., Clarke S.C. (2012). Prevalence of *Streptococcus pneumoniae* serotypes causing invasive and non-invasive disease in South East Asia: A review. Vaccine.

[24] Srifeungfung S., Tribuddharat C., Comerungsee S., Chatsuwan T., Treerauthanaweeraphong V., Rungnobhakhun P., Nunthapisud P., Chokephaibulkit K. (2010). Serotype coverage of pneumococcal conjugate vaccine and drug susceptibility of *Streptococcus pneumoniae* isolated from invasive or non-invasive diseases in central Thailand, 2006–2009. Vaccine.

[25] Phongsamart W., Srifeungfung S., Dejsirilert S., Chatsuwan T., Nunthapisud P., Treerauthaweeraphong V., Rungnobhakhun P., Chokephaibulkit K. (2007). Serotype distribution and antimicrobial susceptibility of *S. pneumoniae* causing invasive disease in Thai children younger than 5 years old, 2000–2005. Vaccine.

[26] Dejsirilert S., Sirinavin S., Sawanpanyalert P., Saengsuk L., Polwichai P., Tienkrim S., Ubonyaem N. A nationwide study on serotypes of invasive strains of pneumococcus in Thailand, 1998–2008. Proceedings of the 7th International Symposium on Pneumococci and Pneumococcal Diseases.

[27] Suwanpakdee D., Samakoses R., Sirinavin S., Kerdpanich A., Simasathien S., Thunyaharn S., Dejsirilert S., Watanaveeradej V. (2010). Invasive pneumococcal disease in Phramongkutklao Hospital 2004–2008: Clinical data, serotype distribution and antimicrobial resistance patterns. J. Med. Assoc. Thai..

[28] Levine S., Dejsirilert S., Sangsuk L., Chantra S., Feikin D.R., Dowell S.F., Olsen S.J. (2006). Serotypes and antimicrobial resistance of *streptococcus pneumoniae* in Thailand 2002–2004. Pediatr. Infect. Dis. J..

[29] Srifeungfung S., Chokephaibulkit K., Tribuddharat C. (2007). Serotypes and antimicrobial susceptibilities of *Streptococcus pneumoniae* isolated from hospitalized patients in Thailand. Southeast Asian J. Trop. Med. Public Health.

[30] Song J.H., Lee N.Y., Ichiyama S., Yoshida R., Hirakata Y., Fu W., Chongthaleong A., Aswapokee N., Chiu C.H., Lalitha M.K. (1999). Spread of drug-resistant *Streptococcus pneumoniae* in Asian countries: Asian Network for Surveillance of Resistant Pathogens (ANSORP) Study. Clin. Infect. Dis..

[31] Song J.H., Jung S.I., Ko K.S., Kim N.Y., Son J.S., Chang H.H., Ki H.K., Oh W.S., Suh J.Y., Peck K.R. (2004). High prevalence of antimicrobial resistance among clinical *Streptococcus pneumoniae* isolates in Asia (an ANSORP study). Antimicrob. Agents Chemother..

[32] Kim S.H., Song J.H., Chung D.R., Thamlikitkul V., Yang Y., Wang H., Lu M., So T.M., Hsueh P.R., Yasin R.M. (2012). Changing trends in antimicrobial resistance and serotypes of *Streptococcus pneumoniae* isolates in Asian countries: An Asian Network for Surveillance of Resistant Pathogens (ANSORP) study. Antimicrob. Agents Chemother..

[33] Baggett H.C., Peruski L.F., Olsen S.J., Thamthitiwat S., Rhodes J., Dejsirilert S., Wongjindanon W., Dowell S.F., Fischer J.E., Areerat P. (2009). Incidence of pneumococcal bacteremia requiring hospitalization in rural Thailand. Clin. Infect. Dis..

[34] Bogaert D., Engelen M.N., Timmers-Reker A.J., Elzenaar K.P., Peerbooms P.G., Coutinho R.A., de Groot R., Hermans P.W. (2001). Pneumococcal carriage in children in The Netherlands: A molecular epidemiological study. J. Clin. Microbiol..

[35] Dagan R., O’Brien K.L. (2005). Modeling the association between pneumococcal carriage and child-care center attendance. Clin. Infect. Dis..

[36] De Lencastre H., Kristinsson K.G., Brito-Avô A., Sanches I.S., Sá-Leão R., Saldanha J., Sigvaldadottir E., Karlsson S., Oliveira D., Mato R. (1999). Carriage of respiratory tract pathogens and molecular epidemiology of *Streptococcus pneumoniae* colonization in healthy children attending day care centers in Lisbon, Portugal. Microb. Drug Resist..

[37] Huang S.S., Finkelstein J.A., Lipsitch M. (2005). Modeling community and individual-level effects of child-care center attendance on pneumococcal carriage. Clin. Infect. Dis..

[38] Sá-Leão R., Tomasz A., Sanches I.S., Nunes S., Alves C.R., Avô A.B., Saldanha J., Kristinsson K.G., de Lencastre H. (2000). Genetic diversity and clonal patterns among antibiotic-susceptible and -resistant *Streptococcus pneumoniae* colonizing children: Day care centers as autonomous epidemiological units. J. Clin. Microbiol..

[39] Yagupsky P., Porat N., Fraser D., Prajgrod F., Merires M., McGee L., Klugman K.P., Dagan R. (1998). Acquisition; carriage, and transmission of pneumococci with decreased antibiotic susceptibility in young children attending a day care facility in southern Israel. J. Infect. Dis..

[40] Pickering L.K., Baker C.J., Kimberlin D.W., Long S.S., American Academy of Pediatrics (2012). [Pneumococcal infections]. Red Book: 2012 Report of the Committee on Infectious Diseases.

[41] Thummeepak R., Leerach N., Kunthalert D., Tangchaisuriya U., Thanwisai A., Sitthisak S. (2015). High prevalence of multi-drug resistant *Streptococcus pneumoniae* among healthy children in Thailand. J. Infect. Public Health.

[42] Piralam B., Prosperi C., Thamthitiwat S., Bunthi C., Sawatwong P., Sangwichian O., Higdon M.M., Watson N.L., Deloria Knoll M., Paveenkittiporn W. (2020). Pneumococcal colonization prevalence and density among Thai children with severe pneumonia and community controls. PLoS ONE.

[43] Hicks L.A., Chien Y.W., Taylor T.H., Haber M., Klugman K.P. (2011). Active Bacterial Core Surveillance (ABCs) Team. Outpatient antibiotic prescribing and nonsusceptible *Streptococcus pneumoniae* in the United States, 1996–2003. Clin. Infect. Dis..

[44] Soeters H.M., von Gottberg A., Cohen C., Quan V., Klugman K.P. (2012). Trimethoprim-sulfamethoxazole prophylaxis and antibiotic nonsusceptibility in invasive pneumococcal disease. Antimicrob. Agents Chemother..

[45] Arason V.A., Kristinsson K.G., Sigurdsson J.A., Stefánsdóttir G., Mölstad S., Gudmundsson S. (1996). Do antimicrobials increase the carriage rate of penicillin resistant pneumococci in children? Cross sectional prevalence study. BMJ.

[46] Nelson K.N., Grijalva C.G., Chochua S., Hawkins P.A., Gil A.I., Lanata C.F., Griffin M.R., Edwards K.M., Klugman K.P., Vidal J.E. (2018). Dynamics of Colonization of *Streptococcus pneumonia* Strains in Healthy Peruvian Children. Open Forum Infect. Dis..

[47] Sutcliffe C.G., Shet A., Varghese R., Veeraraghavan B., Manoharan A., Wahl B., Chandy S., Sternal J., Khan R., Singh R.K. (2019). Nasopharyngeal carriage of *Streptococcus pneumoniae* serotypes among children in India prior to the introduction of pneumococcal conjugate vaccines: A cross-sectional study. BMC Infect. Dis..

[48] Bogaert D., Sluijter M., Toom N.L., Mitchell T.J., Goessens W., Clarke S.C., de Groot R., Hermans P. (2006). Dynamics of pneumococcal colonization in healthy Dutch children. Microbiology.

[49] Lynch J.P., Zhanel G.G. (2010). *Streptococcus pneumoniae*: Epidemiology and risk factors, evolution of antimicrobial resistance, and impact of vaccines. Curr. Opin. Pulm. Med..

[50] Hausdorff W.P. (2002). Invasive pneumococcal disease in children: Geographic and temporal variations in incidence and serotype distribution. Eur. J. Pediatr..

[51] Ghaffar F., Barton T., Lozano J., Muniz L.S., Hicks P., Gan V., Ahmad N., McCracken G.H. (2004). Effect of the 7-valent pneumococcal conjugate vaccine on nasopharyngeal colonization by *Streptococcus pneumoniae* in the first 2 years of life. Clin. Infect. Dis..

[52] Douglas R.M., Paton J.C., Duncan S.J., Hansman D.J. (1983). Antibody response to pneumococcal vaccination in children younger than five years of age. J. Infect. Dis..

[53] Nuorti J.P., Whitney C.G., Centers for Disease Control and Prevention (CDC) (2010). Prevention of pneumococcal disease among infants and children—Use of 13-valent pneumococcal conjugate vaccine and 23-valent pneumococcal polysaccharide vaccine—Recommendations of the Advisory Committee on Immunization Practices (ACIP). MMWR Recomm. Rep..

[54] Doern G.V., Richter S.S., Miller A., Miller N., Rice C., Heilmann K., Beekmann S. (2005). Antimicrobial resistance among *Streptococcus pneumoniae* in the United States: Have we begun to turn the corner on resistance to certain antimicrobial classes?. Clin. Infect. Dis..

[55] Dejsirilert S., Overweg K., Sluijter M., Saengsuk L., Gratten M., Ezaki T., Hermans P.W. (1999). Nasopharyngeal carriage of penicillin-resistant *Streptococcus pneumoniae* among children with acute respiratory tract infections in Thailand: A molecular epidemiological survey. J. Clin. Microbiol..

[56] Lee N.Y., Song J.H., Kim S., Peck K.R., Ahn K.M., Lee S.I., Yang Y., Li J., Chongthaleong A., Tiengrim S. (2001). Carriage of antibiotic-resistant pneumococci among Asian children: A multinational surveillance by the Asian Network for Surveillance of Resistant Pathogens (ANSORP). Clin. Infect. Dis..

[57] Chokephaibulkit K., Srifuengfung S., Mingbanjerdsuk J., Tosasuk K., Vanprapar N., Chearskul S., Dhiraputra C. (2000). Evaluation of susceptibility status of invasive pneumococcal isolates to various antibiotics and risk factors associated with invasive penicillin-nonsusceptible pneumococcal infection: Bangkok 1997-1998. Southeast Asian J. Trop. Med. Public Health.

[58] Pancharoen C., Chongthaleong A., Reinprayoon S., Thisyakorn U. (2001). Invasive pneumococcal infection and drug-resistant *Streptococcus pneumoniae* in Thai children. J. Med. Assoc. Thai..

[59] Centers for Disease Control and Prevention (2009). Active Bacterial Core Surveillance Report, Emerging Infections Program Network, *Streptococcus pneumonia*. http://www.cdc.gov/abcs/reports-findings/survreports/spneu09.pdf.

[60] Jacobs M.R., Good C.E., Windau A.R., Bajaksouzian S., Biek D., Critchley I.A., Sader H.S., Jones R.N. (2010). Activity of ceftaroline against recent emerging serotypes of *Streptococcus pneumoniae* in the United States. Antimicrob. Agents Chemother..

[61] Bogaert D., De Groot R., Hermans P.W. (2004). *Streptococcus pneumoniae* colonisation: The key to pneumococcal disease. Lancet Infect. Dis..

[62] Lee H., Nahm M.H., Burton R., Kim K.H. (2009). Immune response in infants to the heptavalent pneumococcal conjugate vaccine against vaccinerelated serotypes 6A and 19A. Clin. Vaccine Immunol..

[63] Swenson J.M., Hill B.C., Thornsberry C. (1986). Screening pneumococci for penicillin resistance. J. Clin. Microbiol..

[64] Robbins J.B., Austrian R., Lee C.J., Rastogi S.C., Schiffman G., Henrichsen J., Mäkelä P.H., Broome C.V., Facklam R.R., Tiesjema R.H. (1983). Considerations for formulating the second-generation pneumococcal capsular polysaccharide vaccine with emphasis on the cross-reactive types within groups. J. Infect. Dis..

[65] Sanz J.C., Culebras E., Ríos E., Rodríguez-Avial I., Wilhelmi I., Ramos B., Ordobás M., Picazo J.J. (2010). Direct serogrouping of *Streptococcus pneumoniae* strains in clinical samples by use of a latex agglutination test. J. Clin. Microbiol..

[66] Whitney C.G., Pilishvili T., Farley M.M., Schaffner W., Craig A.S., Lynfield R., Nyquist A.C., Gershman K.A., Vazquez M., Bennett N.M. (2006). Effectiveness of seven-valent pneumococcal conjugate vaccine against invasive pneumococcal disease: A matched case-control study. Lancet.

[67] Yu X., Gray B., Chang S., Ward J.I., Edwards K.M., Nahm M.H. (1999). Immunity to cross-reactive serotypes induced by pneumococcal conjugate vaccines in infants. J. Infect. Dis..

[68] Poolman J., Frasch C., Nurkka A., Käyhty H., Biemans R., Schuerman L. (2011). Impact of the conjugation method on the immunogenicity of *Streptococcus pneumoniae* serotype 19F polysaccharide in conjugate vaccines. Clin. Vaccine Immunol..

[69] National Committee for Clinical Laboratory Standards (1997). Performance Standards for Antimicrobial Disk Susceptibility Tests.

[70] National Committee for Clinical Laboratory Standards (1997). Methods for Dilution Antimicrobial Susceptibility Tests for Bacteria that Grow Aerobically.

[71] World Health Organization and Centers for Disease Control and Prevention (U.S.) (2011). Chapter 8 Identification and Characterization of *Streptococcus pneumoniae*. Laboratory Methods for the Diagnosis of Meningitis caused by Neisseria meningitides, Streptococcus Pneumoniae, and Haemophilus Influenzae, WHO Manual.

[72] CLSI (2012). Performance Standards for Antimicrobial Susceptibility Testing.

